# Early intervention for eating disorders

**DOI:** 10.1097/YCO.0000000000000963

**Published:** 2024-09-18

**Authors:** Regan Mills, Lucy Hyam, Ulrike Schmidt

**Affiliations:** a Department of Clinical, Education and Health Psychology, University College London; b Centre for Research in Eating and Weight Disorders, Department of Psychological Medicine, King's College London, Institute of Psychiatry, Psychology and Neuroscience; c Eating Disorders Outpatient Service, Maudsley Hospital, South London and Maudsley NHS Foundation Trust, London, United Kingdom

**Keywords:** anorexia nervosa, bulimia nervosa, early intervention, eating disorders, emerging adulthood

## Abstract

**Purpose of review:**

Research on early intervention for eating disorders has started to gain traction and examples of this in practice are increasing. This review summarizes findings over the past 3 years, focusing on the clinical effectiveness of early intervention in practice and the barriers and facilitators to its implementation.

**Recent findings:**

Recent developments in early intervention for eating disorders can be divided into three broad themes: research that has examined the efficacy of early intervention pathways in practice, research that has informed understanding of the target patient groups of early intervention (via clinical staging models, e.g.), and research that has suggested new ways to progress early intervention, towards becoming a standard part of best practice care.

**Summary:**

Early intervention pathways have shown promising clinical outcomes and are viewed positively by patients, clinicians and other stakeholders. However, more robust trials of their efficacy, effectiveness and cost-effectiveness are needed. Additionally, barriers to early intervention have been identified (e.g. delayed help-seeking); research must now develop and evaluate strategies to address these. Finally, the early intervention models in practice are underpinned partly by clinical staging models for eating disorders, which require further development, especially for eating disorders other than anorexia nervosa.

## INTRODUCTION

Eating disorders typically develop during adolescence and emerging adulthood, from the mid-teens to mid-twenties [1,2]. This is a key developmental time marked by rapid brain development and significant and often challenging transitions between different environments (home, work, and education) and relationships [3]. Identity exploration and formation also begin during adolescence and gain traction in emerging adulthood, when young people for the first time have the legal and financial means for greater independence from their family of origin. These are dimensions of development that are highly related to the manifestation of an eating disorder [4]. Developing an eating disorder during adolescence/emerging adulthood can, therefore, potentially greatly derail psychosocial development [5,6], and extended periods of malnutrition and stress may lead to problematic alterations in brain structure and function [7].

Despite this, emerging adults’ treatment needs are not met as well as those of adolescents, as they are often for the first time, managing their lives independently and without support and supervision from the family. In addition, in many countries, restricted access to specialized care and poor transition management serve as prominent barriers to timely care for young people at the period of greatest need [8]. As such, the time between illness onset and first treatment (i.e. duration of untreated illness, DUED) often spans years and has been estimated at 2.5 years for anorexia nervosa, increasing to 4.4 years for bulimia nervosa and 6 years for binge-eating disorder (BED). Critically, longer DUEDs have been associated with poorer treatment outcomes [9]. In line with this finding, disease burden peaks at 25–29 years for women and 30–34 years for men [10]. Currently, of the individuals who seek and complete eating disorder treatment, only ∼50% will achieve a full recovery [11,12^▪▪^].

Early intervention for eating disorders therefore provides a window of opportunity to target maladaptive thought patterns and behaviours before they become entrenched, to help facilitate a full recovery and prevent lasting consequences on health and life trajectories [13^▪^]. early intervention has been defined broadly as ‘the detection of illness at the earliest possible point during the course of a diagnosable disorder, followed by the initiation of stage-specific, tailored or targeted evidence-based treatment, which is adapted and sustained for as long as necessary and effective’ [14]. early intervention is distinct from but on a spectrum with prevention. Although early intervention focuses on emerging disease, prevention aims to intervene before a disease reaches the diagnostic threshold, typically by targeting modifiable risk and/or protective factors [15]. Prevention programs may be selective (for a high-risk subgroup), targeted (for individuals with early signs of an eating disorder), or universal (for the whole population). early intervention, on the other hand, can be understood as a service model that consists of complex, integrated, and multidisciplinary activities and interventions, designed to achieve effective well coordinated care at the earliest opportunity. The latter point is critical, as evidence suggests that treatment within the first 3 years of an eating disorder may result in a higher chance of recovery [9].

It should be noted, however, the term ‘early intervention’ is also sometimes used to describe stage-specific interventions. In what follows we will try to distinguish between the two, as appropriate.

The aim of this review is to summarize recent developments in early intervention for eating disorders, which can be divided into research that has: assessed the effectiveness of early intervention in practice; attempted to advance our understanding of early intervention target patient groups (e.g. application of clinical staging models and qualitative research), and; summarized new research directions for early intervention. 

**Box 1:**
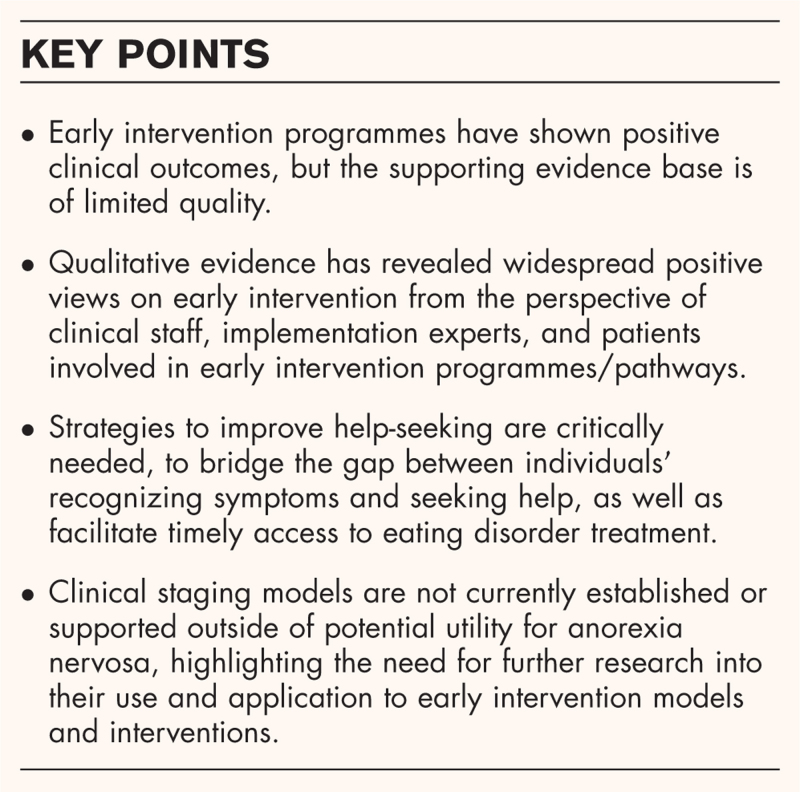
no caption available

## EARLY INTERVENTION FOR EATING DISORDERS IN PRACTICE

**FIGURE 1:**
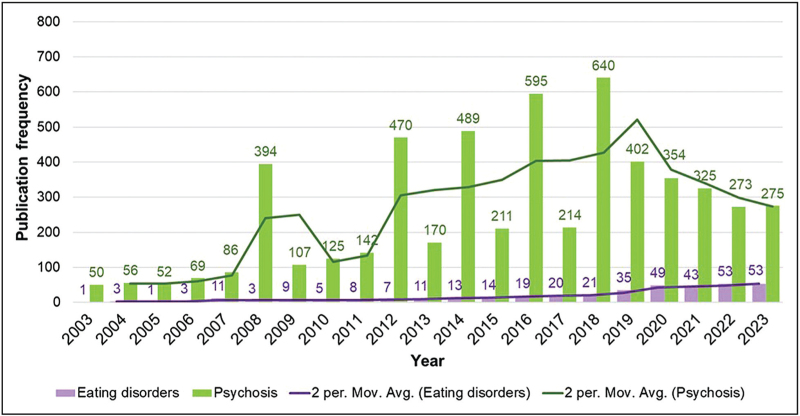
Comparison of frequency of publications on early intervention between psychosis and eating disorders – Web of Science. Comparison and growth of research outputs (Web of Science) in early intervention for psychosis and eating disorders between 2003 and 2023, conducted May 2024. Search terms: (‘Early intervention’ AND ‘eating disorders’); (’Early intervention’ AND ‘psychosis’), with moving average trendlines displayed.

Over the past 10 years, research interest on early intervention for eating disorders has increased substantially but has lagged behind early intervention in other psychiatric disorders. However, traction is growing in research and in clinical practice. Figure 1 demonstrates the frequency per year of retrieved academic publications in a search for (’early intervention’ AND ‘eating disorders’) on Web of Science, over a 20-year period between 2003 and 2023. For eating disorders, output from 2023 is over four-fold that of 2013 (53 publications compared with 11, respectively). In comparison, a similar search for (’early intervention’ AND ‘psychosis’) returns 170 results for 2013 compared with 275 for 2023. This visualization reflects a delay in research activity of arguably over 20 years, with the frequency of research articles on early intervention and eating disorders in 2023 roughly matching those retrieved for psychosis in 2003/2004. This is not surprising given that eating disorder research is substantially less well funded than that of other psychiatric disorders including psychosis, and is less likely to be published in top-ranking journals and is thus less visible [16–18]. This is despite eating disorders having a similar prevalence and burden [10].

Over the past 10 years, various groups have sought to assess the clinical effectiveness of early intervention for eating disorders. Accordingly, a recent rapid systematic review identified 14 publications that assessed either early intervention services or treatment programmes for young people during the early years of their eating disorder (i.e. DUED ≤3 years) [19^▪▪^]. Seven publications reported data from three studies, evaluating aspects of the First Episode Rapid Early Intervention for Eating Disorders (FREED) service model in the UK, whereas one study reported findings from the Emerge-ED programme (modelled on FREED) in South Australia. The remaining studies focused on assessing outcomes from a range of treatments and treatment settings, in children or adolescents with anorexia nervosa only and who had a short illness duration (<3 years). Treatments included family-based treatment (FBT), home-based treatment after initial hospitalization or on an outpatient basis, and routine outpatient or day treatments.

For both FREED and Emerge-ED, the authors reported positive clinical outcomes. Participants experienced mean reductions in eating disorder symptomology, psychological distress and a reduced DUED, as well as more favourable treatment uptake and wait times for assessment and treatment when compared with a retrospective treatment-as-usual (TAU) cohort (e.g. 3.6 versus 6.7 weeks to assessment and 8 versus 20.8 weeks to treatment for FREED and TAU groups, respectively [20]) [21,22]. This finding is of crucial clinical relevance given that a recent meta-analysis found, when compared with other moderators of treatment outcomes, waiting lists were associated with the highest mortality rates in people with anorexia nervosa [12^▪▪^]. In the studies assessing either outpatient treatments or multidisciplinary interventions in young people with anorexia nervosa of short illness duration, the results were positive, with BMI increasing in all studies [23–25]. There were also reductions in eating disorder symptomology and improved global functioning and psychological impact scores over time.

Hamson *et al.*[19^▪▪^] noted that all the included studies had high risk of bias, primarily because of a lack of comparative data, confounding factors, and missing participant data. It is arguable whether all of the studies testing interventions in children and adolescents with illness duration of less than 3 years should be seen as delivering early intervention, as some included young people who had a previous inpatient admission for their eating disorder (i.e. those who had intensive or prolonged service involvement). Nonetheless the current evidence-base for early intervention service models, as well as the developmentally and illness-stage appropriate interventions within them, demonstrates the positive impacts of different early intervention programmes and interventions in real-world settings, where it may be challenging to collect high-quality data from service users. In a consensus framework [13^▪^] for progressing early intervention for eating disorders, a key research recommendation was the continued evaluation of early intervention programmes and associated developmentally and illness-stage appropriate interventions, including randomized controlled trials and including longer term follow-ups. For early intervention service models, which focus on delivery of well coordinated multidisciplinary care, fidelity assessments (comparable with the First Episode Psychosis Services Fidelity Scale [26]) may also be needed, particularly during the scaling up of such models and when assessing their sustainability [27]. These assessments may help develop a more fine-grained understanding of what specific components of these complex programmes are feasible (within the context of resource constraints), and what components impact on treatment outcomes.

In a second rapid review, Koreshe *et al.*[28^▪▪^] identified 37 articles on early intervention for eating disorders. This larger pool of early intervention studies (compared with 14 from Hamson *et al.*'s review) likely reflects the lack of consensus regarding what constitutes early intervention for eating disorders and the unclear distinction between early intervention and prevention programmes. Of the included early intervention studies, a significant proportion were delivered online. These ranged from combined screening and early intervention programmes such as ‘ProYouth’, to web-based interventions designed to increase treatment adherence and motivation to change [29,30]. The preassessment ‘MotivATE’ programme, for example, increased attendance at an eating disorder appointment almost 10-fold in intervention completers compared with those who did not engage with the intervention [31]. This shift towards online delivery reflects the increasing need to develop early intervention programmes that are both cost-effective and accessible to different populations, in a setting where human and financial resources for eating disorder treatment and research are limited [18,32]. Although there is no comparative cost-analysis of early intervention programmes to date, initial evidence suggests long-term cost savings when intervening earlier in the course of an eating disorder (e.g. approximately £10 million in health service cost-savings for FREED between 2016 and 2023) [33]. Societal cost-savings are likely much larger.

## BARRIERS AND FACILITATORS OF EARLY INTERVENTION FOR EATING DISORDERS

Early intervention requires treatment access. International data, however, suggests only a fifth of people with an eating disorder access treatment [34]. Recent articles examining the barriers and facilitators of early intervention for eating disorders have identified a range of factors associated with help-seeking that may inform early intervention strategies and work towards improving early treatment-seeking rates. A meta-analysis by Radunz *et al.*[35^▪▪^], for example, identified 24 unique variables related to help-seeking. Notably, only two factors, ‘perceived inability of others to provide help’ and ‘denial/failure to perceive eating disorder severity’, were significantly and negatively associated with treatment-seeking behaviour.

Being unable to recognize that there is a problem, downplaying, underestimating or denying the severity of the eating disorder has repeatedly emerged as a prominent barrier to early intervention for eating disorders. In a sample of 137 women with high impairment and elevated eating disorder concerns, Fabry *et al.*[36] found that 85% thought help-seeking would be useful. Only 39%, however, had sought professional help for their own concerns. Barriers reported most frequently were denial and self-reliance (the belief that they should resolve their own problems), both of which moderated the association between help-seeking attitudes and behaviours. This finding was replicated by Radunz *et al.*[37], who reported a unique association between treatment-seeking and the denial subscale, when investigating the factor structure of eating disorder questionnaires in a group of high-risk women. In another recent study, 80 participants from the Emerge-ED cohort were asked for their views on barriers to treatment-seeking. The most cited barrier was ‘belief that my problem is not bad enough’, reflective of an underestimation of illness severity [38^▪^].

To address barriers such as impaired illness recognition and low motivation to change, several systematic reviews identified support and encouragement from friends and family as a key facilitator of early intervention for eating disorders [39,40^▪^,^41^]. In a pilot trial, Wade *et al.*[42] offered guided self-help (GSH) to families on waitlists for FBT for children with anorexia nervosa. Over 12 sessions, children experienced reductions in eating disorder behaviours and a mean weight gain of 6 kg, while parents reported increased knowledge, skills, and confidence in managing anorexia nervosa. Of 187 eligible families on the waitlist, however, only 13% expressed interest in participation. Strategies to improve engagement with parents in early intervention, therefore, seem key, although the level of parental involvement may vary for emerging adults, who may want to balance receiving support from caregivers with an increased desire for independence and autonomy.

To overcome service and healthcare system-related barriers (e.g. long waitlists and high service demand), several creative, lower cost solutions have been evaluated. As highlighted in a recent review by Mills *et al.*[43^▪^], these include single session interventions, abbreviated treatments (such as GSH), and task-sharing among nonspecialists. Although these interventions have yet to be widely adopted in routine clinical practice (with GSH being offered to 15% of eligible patients in the FREED pathway [44]), there is both scope and a necessity to implement these interventions as accessible, effective, and scalable early intervention treatment options.

## QUALITATIVE EVIDENCE ON EARLY INTERVENTION PROGRAMMES FOR EATING DISORDERS

Recent qualitative research on early intervention in practice has also focused on the perceived facilitators and barriers to early intervention for eating disorders. In addition to exploring the views of patients, Radunz *et al.*[38^▪^] investigated the views of healthcare professionals involved in the delivery of the Emerge-ED programme. One of the major barriers cited by clinicians was that there were more people eligible for the pathway than could be managed. Young people with BED or Avoidant Restrictive Food Intake Disorder (ARFID) diagnoses were, therefore, excluded from the programme. To be given access to Emerge-ED, young people also had to demonstrate that they had not had any prior attempt at evidence-based treatment for eating disorders. This highlights the issue that in publicly funded services, demand for early intervention outstrips resources, disadvantaging those who are not at immediate high medical risk.

Two integrated studies investigated the views and experiences of clinicians involved in the implementation of the FREED early intervention model, and the views of implementation specialists who had facilitated the national roll-out of FREED. Overall, there was strong support for early intervention for eating disorders from both clinicians and implementation specialists, and views that the implementation of FREED had actual and anticipated benefits for patients. A strong practitioner network for implementation support, as well as supportive management and teams, were perceived as critical to the successful implementation of FREED. However, persistent and ongoing workforce issues affected the ability to meet waiting time targets for assessment and treatment, presenting a significant barrier to effective early intervention [45^▪^,^46^,^47^]. These findings offer insights into the implementation of an early intervention service in the ‘real world’.

These qualitative studies predominantly included those central to and responsible for FREED implementation (e.g. FREED ‘Champions’). The views of the wider eating disorder service and staff not focussed on early intervention were missing. This is important because there is sometimes a sense that early intervention unfairly prioritizes milder cases or that early intervention patients are being given ‘a special deal’, with other patients not receiving the same degree of support or attention, potentially leading to tensions within a team [46,47]. However, interviews with implementation specialists with experience of implementing multiple health innovations showed broad consensus in these experts’ views that FREED was an important and scalable intervention with clear benefits for the healthcare system [45^▪^]. Qualitative research with patients treated through the FREED pathway also demonstrates support for this; with quick access to treatment, an early focus on recovery, and knowledgeable, hopeful clinicians and developmentally tailored treatment adaptations cited as beneficial [48]. Thus, there is converging evidence highlighting the importance of tailored early intervention and intervention speed (e.g. ‘meeting the patient where they are’).

## APPLICATION OF STAGING MODELS TO EARLY INTERVENTION FOR EATING DISORDERS

Early intervention service models like FREED are underpinned by clinical staging models, with preliminary evidence suggesting such models may have validity and utility in anorexia nervosa [13^▪^]. The concept of an early stage of eating disorders has been applied to early intervention models including FREED, where early intervention is targeted to DUED 3 years or less and between ages 16 and 25 (however, this criterion also had pragmatic value, to target limited resources to the peak age of onset) [49]. Concerns have been raised regarding the DUED criterion around the potential impact on patients not seen within the FREED pathway [45^▪^,^47^]. Although some evidence supports a staging model for anorexia nervosa, with intervention at the earlier stages of an eating disorder leading to better outcomes [50,51], the generalizability of this model to other eating disorders remains unclear. Further research is needed to understand whether staging paradigms apply to other eating disorders, and whether staging criteria based mainly or only on illness duration are appropriate. Work is in progress to review staging models for eating disorders. A systematic scoping review of the literature confirmed that staging models have been predominantly applied to anorexia nervosa, where research suggests that DUED exceeding 7 years for anorexia nervosa may indicate progression to a persistent eating disorder [52^▪^]. However, these timeframes have still not been established for other eating disorder diagnoses. Another scoping review protocol also seeks to review clinical staging concepts for eating disorders including a broader range of evidence, to specifically inform early intervention models like FREED, and whether the DUED criterion is supported by evidence [53].

Of key interest is a recent review and meta-analysis that broadly provides support for a staging model [12^▪▪^]. Children and adolescents were shown to have the highest recovery rates and lowest chronicity rates for anorexia nervosa, suggesting that neurobiological and psychosocial factors may contribute to development and maintenance of the eating disorder, making eating disorders potentially less receptive to current treatments over time. This suggests that DUED criteria may well be appropriate for a specialized early intervention pathway, allowing for personalized intervention at a critical and developmentally sensitive period.

A protocol paper detailing the 4 year EDIFY consortium research programme describes six integrated workstreams to inform personalized prevention and early intervention for eating disorders [54^▪^]. Of these workstreams, two will focus on understanding recovery trajectories and how eating disorder behaviours and brain responses change from early-stage to late-stage illness, which will also contribute to our knowledge of illness progression and clinical staging in eating disorders. Ultimately, appropriate interventions for all illness stages (i.e. regardless of duration of illness or age) are needed [55,56].

## CONCLUSION

The current review highlights a burgeoning and promising evidence-base for early intervention for eating disorders, with ongoing research into different service models for early intervention, such as FREED and Emerge-ED. Reaching a consensus regarding the definition of early intervention for eating disorders will be an important next step, including defining key pathway parameters such as patient eligibility, treatment type and timing of intervention. The development of international, evidence-based clinical guidelines may in turn help improve model fidelity, as well as guide future research into novel early intervention strategies and their application to heterogeneous patient populations.

## Acknowledgements

*None.*

### Financial support and sponsorship

*This work is supported by the Medical Research Council/Arts and Humanities Research Council/Economic and Social Research Council Adolescence, Mental Health and the Developing Mind initiative as part of the EDIFY programme (grant number MR/W002418/1). US receives salary support from the National Institute of Health Research (NIHR) Biomedical Research Centre (BRC) at the South London and Maudsley (SLaM) NHS Foundation Trust and King's College London (KCL). The views expressed herein are those of the authors and not necessarily those of the NHS, NIHR or Department of Health and Social Care.*

### Conflicts of interest

*L.H. and U.S. are part of the FREED national team, located at South London and Maudsley NHS Foundation Trust Eating Disorder Service. L.H. reports PhD Studentship from Health Foundation, outside the submitted work. The authors report no other conflicts of interest in this work.*

## REFERENCES AND RECOMMENDED READING

Papers of particular interest, published within the annual period of review, have been highlighted as:

▪ of special interest
▪▪ of outstanding interest
